# Fever and Ague

**Published:** 1878-03

**Authors:** 


### HALL'S JOURNAL OF HEALTH.

OUR LEGITIMATE SCOPE IS ALMOST BOUNDLESS: FOR WHATEVER BEGETS PLEASURABLE AND HARMLESS FEELINGS, PROMOTES HEALTH; AND WHATEVER INDUCES DISAGREEABLE SENSATIONS, ENGENDERS DISEASE.

*We aim to show how Disease may be avoided, and that it is best, when sickness comes, to take no Medicine without consulting an educated Physician.*

25TH YEAR. NEW YORK, MARCH, 1878. No. 291.

### FEVER AND AGUE.

IN returning from "The Springs," the Sea-side, and other places of resort during the heats of summer, many families have noticed in the autumual and winter months that more or less of the members, especially the children, are quite unwell at times. In a day or two they get better only to feel worse again, and this annoying process continues till the cold weather has steadily set in. Some persons are regularly "ailing" at intervals of days or weeks. The name given to this form of sickness by common people is "the creeps," as the symptoms come on with a chilly sensation of the hands and feet, or along the back, extending generally over the whole body, when there is sometimes a general shiver or shake—to be followed by a fever during the afternoon and going off with a perspiration during the night. In the Western country this is a process which the person attacked has to go through every twenty-four hours for weeks and months; to be resumed the next year, and the next, until in five or ten or more years, the constitution becomes hardened to it or it wears itself out, provided the unhappy patient does not, in the meantime, take a bad cold and become consumptive, or die more summarily of some more active malady.

There is scarcely a locality within thirty miles of New York where families can remain until the first of Autumn without having the seeds of this hateful malady sown in the system, to fructify on their return home and thus do away with all the good effects of a summer's sojourn in the country. It is not at all likely that this state of things will materially alter in this generation, for the laws of nature are uniform ; but it is desirable to interpose some means of fortifying the system against these attacks by scientific appliances. This is certainly demonstrable and possible ; but to do so satisfactorily, it is necessary to understand the whole subject, which may be made exceedingly interesting and is a matter of personal concern to every one who is in the habit of "going to the country" in the summer time. To have the enjoyment of such a pleasant sojourn constantly clouded with the apprehension of the discomforts of having the "creeps" for an indefinite time on returning to town, is certainly not a pleasant contemplation.

The cause of fever and ague is "miasma," the meaning of which word is *emenation*, a 'rising from,' as it is supposed to come up from the surface of the earth and impregnate the atmosphere, which being breathed into the lungs is taken a few seconds later into the circulation, being intimately mixed with the blood and poisons it, causing it to be thick, sluggish, black and impure. In some situations this miasma is so concentrated, saturating the atmosphere, as it were, consequently thickening the blood more rapidly and to such an extent that it flows at first slowly and at length scarcely moves at all at the extremities, and circulates perceptably only about the heart; and as the blood begins to die the instant it ceases to move, the limbs grow cold, the veins are distended, the fire of life goes out and the man dies—of congestive fever. Some have been known to die in the chill of fever and ague, although generally, fever and ague is not considered any more dangerous than the tooth-ache ; hence in both cases the unfortunate victim has very little of the sympathy of those around him.

The substance of miasma has been considered etherial, as the atmosphere of a miasmatic locality upon chemical analysis, made by different experts and in the most careful manner, has not been found to contain any ingredients, hitherto, which did not belong to a pure and healthful atmosphere. Still, although the miasma could not be detected, it was known to be an entity, an actual thing, and men had to be content with studying its nature, and its effects, and its laws, by observation on its modes of action, then recording the facts observed and deducing the laws of its action therefrom. The first name given to it was "marsh miasm," because the effects were observed in the most marked manner in the neighborhood of marshes, of low flat damp lands where vegetation was rank.

It was next observed that the sickness arising from marsh miasm did not occur in cold weather; another step forward was then made, that miasm was peculiar to damp soils, and that heat was necessary for its production. But the effects of miasm were not observed on the sea-shore, although there was dampness and heat and a flat surface. The reason must be because it was sandy; there was no vegetation, hence another element was essential to miasm. There must not only be dampness and heat but there must be vegetation, and when it was later observed that miasmatic diseases were more general and malignant in the Fall of the year, and that was the season when vegetation began to decay and die and decompose, the concatenation was complete and the full idea was expressed in the proposition:—Malaria is an emenation from decaying vegetation in warm weather, hence miasm was caused by vegetable decomposition—such decomposition requiring moisture and heat.

So much for the nature and cause of miasm. Its effects were from time to time noticed as originating in man, diarrhœa dysentery, and all forms of fevers. Its laws of action were next investigated, observation proved it milder in the Spring, more malignant in the Autumn. There was vegetation enough in the Spring and moisture enough, but not sufficient heat in our latitude to cause vegetable decomposition.

It was next observed that persons exposed in miasmatic localities in the night, suffered more than those exposed in the daytime. For fifty years previous to the discovery of gold in California it was known among the commanders of vessels that sailors might go ashore in certain tropical climes in the day-time, but to pass a night on shore was certain death. The more intelligent adventurers who first went to California via the Isthmus of Panama made practical use of this fact, and began the passage early in the day so as to get to the higher points of land before night came on. The immediate cause of the fatal attack of illness to Bishop Potter in his visit to California was inattention to this fact, for he left the ship to perform a marriage cerimony, remained on shore during the night, was soon attacked with a new form of disease and lived just long enough to land at San Francisco.

Old Charleston merchants will remember that while it was considered death for them to sleep in the city during the summer for a single night, habitually rode into the city to transact business in the middle of the day. Twenty years ago the doorways and steps of public buildings in Rome were crowded with sleepers in harvest-time. They were the men who worked in the Pontine marshes during the day-time; they knew it was death to sleep there at night.

Without narrating each particular step in the discovery of the additional laws of miasm, suffice it to say that in *ordinary* localities the effects of miasm were found to be more decided in the hours including sun-rise and sun-set, and that at other times it was almost innoxious. It was very natural then to enquire why was it most hurtful at sun-rise and sun-set to remain in a miasmatic locality? It must be because it was most concentrated at that time; there was more of it in a given amount of air breathed into the lungs. Cold condenses all atmospheres; heat rarifies, expands and sends upward. The heat of the day generated the miasm from the damp decaying vegetation and it rose rapidly towards the clouds; but when the sun began to decline the atmosphere became cooler, more heavy, fell towards the surface and settled within a few feet of it, that layer next the earth being most malignant, and every foot higher the less so. It is known that when a traveler with a dog entered the Grotto del Cano, the dog died while the owner remained uninjured, he being several feet higher, the gas causing death to the dog being so much more concentrated than on the ground. It is known that a man lying down in a poppy-field will die before the morning, at certain seasons; but if he works in it, his standing up enables him to breathe a less compact layer of air. At sun-rise the atmosphere begins to warm and the miasm to ascend, and in the course of an hour it has ascended higher than the head and hence is not taken into the lungs. At mid-day it has gone to the heavens; at midnight it lies immediately on the surface, in each case not breathed into the lungs by a man on his feet.

Now just at this point, a practical and important lesson was to be learned, which for actual practical results in proportion to the expense and labor and trouble, is scarcely second to any other in the whole range of sanitary science; not new, but too simple to command any special general attention. If the heat from the sun, by a general law of nature, so rarifies the miasmatic air as to make it innoxious, artificial heat must do the same thing. If a man will keep a brisk fire burning in his family room for the hour or two including sun-rise and sun-set, and will remain in that room during that time, it will be an absolute exemption from all autumnal diseases, and from cholera itself, other things being equal, for cholera is known to make its greatest ravages where common epidemics prevail in ordinary times, such as fevers, dysentery and diarrhœa and cholera is only an aggravated diarrhœa, as yellow and congestive fevers are the exageration of common fever and ague.

The dreadful ship-fever, jail-fever and the epidemics that occur in crowded vessels arise always from the decay of vegetable matter in the hold of vessels ; the wood of which the vessel is composed being in a state of constant dampness and inevitable decay. Now as there can be no decay where there is dryness, and heat makes dry there is only one way to disenfect a vessel to make it healthy. Empty it ; make it dry as a powder-horn, by stoves or by the more expeditious and less expensive method of introducing heated air into it from a steam engine. A vessel may be frozen up and thus made healthy ; but it is only temporary ; the miasm was only condensed and will make up to all its virulence, as did the viper in the fable, as soon as it is warmed. Heat, on the contrary, rarifies the miasm and sends it to the clouds and, by its drying effects, prevents ts renewal.

The writer spent some years of his life in various malarious countries, and acting in the light of the above principles was never sick an hour in any of them, where he travelled on horse-back in the heats of mid-summer days by the pestiferous vapors of the bayous and visiting the sick at mid-night wherever and whenever called ; but at sun-rise and sun-set, in the heats of July, he was by a blazing fire in his own house, or secured one if abroad. And he can name families in the West in districts where Fever and Ague was universal, except in a solitary house here and there where the friendly fire was started at sun-rise and sun-set, in the family room ; and the breakfast was eaten before going outside the door, and the supper taken at sun-down, the excitement of the circulation caused by the meal, and its strengthening effects on the system, helping to fortify it against the attacks of malarious influences.

But within a year it has been announced as a discovery made by a physician in Chicago, and by a lady in France, and by her communicated to the Academy of Sciences, that the cause of epidemic Fever and the Autumnal diseases was discovered to be a living thing, the gentleman calling it vegetative, a Sporule; the lady asserts it to be an entozon, a breathing animal.

But it is curious to observe that this Sporule or entozon is under the identical laws, supposed to belong to miasm; that heat destroys it; cold benumbs it; that it is most vigorous in its ill effects in the system in the cool of the evening and the morning, and that it is only found in marshy places, in warm weather. Their existence are said to be made visible by the microscope—are seen in the saliva and attached to the inner portion of the mouth; and that if an atmosphere containing them is taken to a distance where it is not naturally existing, and is breathed by a person in health, that person in a few days has Fever and Ague.

In regard to the treatment for Fever and Ague, I differ widely from those who trust implicitly to Quinine. This will break the chills, and if long continued, the health also. The better plan is to cure the disease, and not to smother it.

